# Effects of Exposure to Tobacco Cigarette, Electronic Cigarette and Heated Tobacco Product on Adipocyte Survival and Differentiation In Vitro

**DOI:** 10.3390/toxics8010009

**Published:** 2020-02-05

**Authors:** Zoi Zagoriti, Mohamed A. El Mubarak, Konstantinos Farsalinos, Stavros Topouzis

**Affiliations:** 1 Laboratory of Molecular Biology and Immunology, Department of Pharmacy, University of Patras, 26504 Patras, Greece; zoizag@upatras.gr (Z.Z.); kfarsalinos@gmail.com (K.F.); 2 Laboratory of Pharmacokinetics, Department of Pharmacy, University of Patras, 26504 Patras, Greece; sheikho@upatras.gr; 3 Laboratory of Molecular Pharmacology, Department of Pharmacy, University of Patras, 26504 Patras, Greece

**Keywords:** cigarette smoke, heated tobacco products, electronic cigarettes, nicotine, extracts, beige adipocytes, 3T3-L1 cells

## Abstract

Cigarette smoking (CS) causes significant morbidity worldwide, attributed to the numerous toxicants generated by tobacco combustion. Electronic cigarettes (ECIG) and heated tobacco products (HTP) are considered alternative smoking/vaping products that deliver nicotine through an inhaled aerosol and emit fewer harmful constituents than CS. However, their long-term impacts on human health are not well established. Nicotine exposure has been linked to lipolysis and body weight loss, while smoking has been associated with insulin resistance and hyperinsulinemia. Enhanced function of beige (thermogenic) adipocytes has been proposed as a means to reduce obesity and metabolic disorders. In this study, we compared the effect of extract-enriched media via exposure of culture medium to CS, HTP aerosol, and ECIG aerosol on the viability and the differentiation of 3T3-L1 pre-adipocytes to beige adipocytes. Only CS extract caused a decrease in cell viability in a dose- and time-dependent manner. Furthermore, relative lipid accumulation and expression levels of the adipocyte markers *Pgc-1α*, *Ppar-γ* and *Resistin* were significantly decreased in cells exposed to CS extract. Our results demonstrate that CS extract, in contrast to HTP and ECIG extracts, significantly impairs differentiation of pre-adipocytes to beige adipocytes and may therefore impact significantly adipose tissue metabolic function.

## 1. Introduction

Cigarette smoke (CS) is a complex mixture of nearly 7000 chemical compounds, including volatile constituents that remain in the gas phase, such as aldehydes, acrolein, and carbon monoxide (CO) and constituents that are detected in the particulate phase, comprising nicotine and other alkaloids, tobacco-specific nitrosamines (TSNAs), and polycyclic aromatic hydrocarbons (PAHs) [1]. Sixty nine smoke components constitute strong or weak carcinogens and are present in cigarette mainstream and sidestream smoke [2]. Smoking constitutes a major risk factor for lung cancer, manifested as a greater propensity of smokers to develop squamous cell carcinoma and small cell lung cancer than adenocarcinoma [3]. Numerous epidemiological studies have also shown that long-term smoking is among the best established risk factors for many non-pulmonary cancers, including pancreatic [4] and colorectal cancer [5]. In addition to the apparent role on human cancer, smoking has been implicated in cardiovascular [6,7], metabolic [8], and respiratory diseases [9]. In the adipose tissue, nicotine has been reported to induce lipolysis, leading to body weight loss [10], while smoking has been associated with insulin resistance and hyperinsulinemia [11,12], therefore altering the metabolic function of this tissue.

Electronic cigarettes (ECIG) and heated tobacco products (HTP) have been designed to deliver nicotine through an inhaled aerosol, without combusting tobacco. Over the last few years, these nicotine products have been suggested as less harmful alternatives to conventional cigarettes (i.e., “reduced risk” products) and have entered the market as potential smoking cessation tools. Due to the lack of sidestream emissions (in the case of ECIGs) or presence of minimal sidestream emissions (in the case of HTPs) in indoor environments compared with conventional cigarettes, these products are more socially acceptable and subjected to less stringent regulation than tobacco cigarettes [13]. However, because of their novelty, there is inadequate scientific evidence on the impact of long-term use of these products. Analytical studies have identified toxic substances that are classified as carcinogens, such as aldehydes and TSNAs, in the ECIG aerosol, but in substantially lower levels than found in CS [14,15,16]. Similarly, following the assumption of temperature-dependent production, these harmful constituents were detected in reduced amounts in the aerosol of HTP [17,18,19,20], while the nicotine levels delivered are only slightly lower than those of conventional CS [20,21]. In addition to the chemical composition, the physical properties of the smoke or aerosol can greatly influence the biological outcome of the exposure. A case-in-point are ultrafine particles (< 100 nm), which are of great toxicological importance, because of higher deposition in the respiratory tract. Second-hand emissions of CS from conventional cigarettes contain much higher levels of ultrafine particles than those from HTP and ECIG [22]. Additionally, infants and children were found to be more susceptible than adults, as they received the largest quantities of particles per kg of body weight that could be translocated to the brain via the olfactory bulb [22]. 

At the preclinical level, an increasing number of in vitro studies have been conducted in order to assess the cytotoxic, inflammatory, and oxidative effects of ECIG and HTP. However, given the variety of products, flavoring additives, and functional characteristics, as well as the diversity of exposure strategies and target cells, the results are difficult to interpret. In general, the majority of studies using airway epithelial cells have revealed that the exposure to ECIG and HTP aerosol can induce cytotoxicity and production of pro-inflammatory mediators and reduced antioxidant capacity, but to a lesser extent than CS [23,24].

There is limited information regarding the potential role of these alternative smoking/vaping products on adipose tissue and adipocyte function. Beige adipocytes, also called brown-like or brite (brown-in-white), are found scattered within the white adipose tissue depots of rodents and humans. Beige adipocytes share common morphological and metabolic features with both brown and white adipocytes, but they possess a distinct gene expression profile [25]. In response to various stimuli activating β-adrenergic receptor or peroxisome proliferator-activated receptor-γ (*Ppar-γ*), beige adipocytes acquire a brown adipocyte-like phenotype and an inducible thermogenic capacity, both gradually reversing upon stimulus withdrawal [26,27]. Since classical brown adipocytes expend stored energy to generate heat, this adaptive form of thermogenic potential observed in beige adipocytes could lead to an effective therapeutic means against obesity and related metabolic diseases [28].

The mechanism of non-shivering thermogenesis is mediated by uncoupling protein 1 (*Ucp1*), which disrupts the electrochemical gradient across the inner mitochondrial membrane, thus uncoupling the respiratory chain from adenosine triphosphate (ATP) synthesis and resulting in heat generation [29]. Although all thermogenic adipocytes are *Ucp1*-positive, *Ucp1* is highly expressed in brown adipocytes and in low basal levels in beige adipocytes [25]. Given the crucial role of *Ucp1* in thermogenic activity and ATP synthesis, transcription of *Ucp1* is regulated by multiple transcription factors, including *Ppar-γ*, PR domain containing 16 (Prdm16), and peroxisome proliferator-activated-receptor gamma coactivator-1 alpha (*Pgc-1α*) [30].

Taking into account the lack of research on the effects of ECIGs and HTPs on adipocytes, the dual purpose of this study was to: (a) establish a method to enrich cell growth medium in products contained in CS and in ECIG and HTP aerosols, in order to (b) evaluate and compare the effect of CS, HTP and ECIG aerosol extracts on the viability and the differentiation of 3T3-L1 pre-adipocytes towards beige adipocytes.

## 2. Materials and Methods

### 2.1. Preparation of Extract-Enriched Media

The administration of CS, HTP, and ECIG aerosol in cell cultures required the preparation of extract-enriched media (called “extracts” from now on) by exposure of the culture medium (Dulbecco’s modified Eagle’s medium; DMEM, Biosera, Nuaillé, France) to each aerosol. These aqueous extracts of CS, ECIG vapor and HTP aerosol were collected using a set of 2 impingers connected in series and attached to a custom-made smoking machine, programmable for puff number, duration and volume. The puffing protocol involved 2-s puffs with 30-s puff intervals and a puffing volume of 40 mL. Briefly, mainstream CS, HTP aerosol, ECIG aerosol, or air (as control) was drawn through the impingers and dissolved into DMEM culture medium contained in the 2 impingers (20 mL/impinger), generating a condensed liquid form of each aerosol.

CS extract was produced by smoking three reference 1R6F cigarettes (University of Kentucky, Lexington, KY, USA), containing 8.6 mg tar and 0.7 mg nicotine per cigarette. Cigarettes were combusted to a fixed butt length (the length of the filter overwrap paper plus 3 mm), with ventilation occlusion (~9 puffs/cigarette, 27 puffs in total). A commercially available HTP (iQOS, Philip Morris International Global Services Inc., New York, NY, USA) was purchased and four tobacco sticks of regular flavor were used for the extract generation (12 puffs/stick, 48 puffs in total).

The ECIG device used for the extract preparation was composed of a EVIC VTC battery device (Joyetech Co. Ltd, Shenzhen, China) and a 2 mL Zenith atomizer (Innokin, Shenzhen, China) coupled with a 0.8 Ohm stainless steel resistance atomizer head. The device power was set at 16 W. The e-cig liquid was custom made at the laboratory, composed of 49.4% *w*/*w* propylene glycol, 49.4% *w*/*w* glycerol and 1.2% *w*/*w* nicotine (no flavor was added). The liquid tank was filled with 2 mL of the above liquid and the aerosol of 48 puffs was collected in the impingers.

The blank extracts (called ambient air extracts from now on) were generated by performing 48 puffs of ambient air that passed through the set of impingers.

### 2.2. Analysis of Nicotine with Liquid Chromatography–Tandem Mass Spectrometry (LC–MS/MS)

The concentration of nicotine in each extract was determined, so that cells would be treated at equivalent amounts of nicotine from each extract, as an equalizing factor. Analysis was performed using liquid chromatography–tandem mass spectrometry (LC–MS/MS). The LC-MS/MS analyses were carried out with Waters Alliance HT 2795 liquid chromatography system and Micromass Quattro Micro tandem mass spectrometry (MS) system. An accurate and sensitive method for the quantification of analyte in extracts was developed and validated. The Micromass Quattro Micro tandem MS system (Waters, Milford, MA, USA) was equipped with an electrospray ion source and was operated in the positive ion mode. For the optimization of mass spectrometer conditions, a standard solution [500 ng/mL of nicotine and 4-(Methylnitrosamino)-1-(3-pyridyl)-1-butanol (NNAL) purchased from Sigma-Aldrich Chemie GmbH, Taufkirchen, Germany internal standard (IS) in acetonitrile] was directly introduced at a flow rate of 30 μL/min. The parameters were optimized as following: source temperature 100 °C, desolvation temperature 400 °C, desolvation gas flow 500 L/h and cone gas flow 50 L/h. The capillary voltage was set at 3.5 kV, while the multiplier was set at 650 V and argon was used as the collision gas. Quantification of nicotine and IS were achieved (Triple Quadrupole Mass Analyzer) by high sensitivity and specific Multiple Reaction Mode (MRM) scan, using product-ions *m*/*z* 163.3 > 130.1 and 210.6 > 93.14, respectively. Moreover, the analyte was separated by reversed-phase chromatography using a Phenomenex (Gemini 3 μm, NX- C18, 110Å, 150 × 2 mm) column equipped with an additional guard column (SecurityGuard ULTRA cartridge, Gemini C18), in the HPLC system consisted of a quartenary pump (Waters, Milford, MA, USA) accompanied by an autosampler, a degasser and a column oven/cooler. Therefore, analyte separation was achieved in a gradient elution of solvent A (20 mM Ammonium bicarbonate in H_2_O purchased from Sigma-Aldrich Chemie GmbH, Taufkirchen, Germany) and solvent B (acetonitrile). A gradient elution was started with 30% of solvent B, then increased with a linear increment to 80% until 2.5 min and maintained for 0.5 min. The elution was then decreased to 30% of solvent B in 3.1 min, and this composition was maintained until the end of the run (8 min). The column temperature was set at 40 °C throughout all experiments, whereas the sample temperature was kept at 15 °C. Fifty microliters volume of injection was delivered with a constant flow rate 0.25 mL min^−1^. The method was found to be linear in the concentration range of 1.0–200.0 ng/mL for nicotine. All solvents used were LC-MS grade from Fisher Scientific, Loughborough, UK and were filtered through 0.22 μm filters (Titan Membrane, Merck Millipore, Burlington, MA, USA). Data acquisition and processing were performed using the Masslynx (v. 4.1, Waters Corporation, Milford, MA, USA, 2005) software program.

### 2.3. 3T3-L1 Cell Culture

Mouse embryo 3T3-L1 pre-adipocytes (ATCC^®^ CL-173^™^, ATCC, Manassas, VA, USA) were grown in DMEM with high glucose content (4.5 g/L), supplemented with 10% *v*/*v* fetal bovine serum (Gibco, Thermo Fisher Scientific, Waltham, MA, USA),100 units/mL penicillin and 100 µg/mL streptomycin (Biosera, Nuaillé, France). Cells were maintained at 37 °C, with 5% CO_2_ in a humidified incubator.

### 2.4. Cell viability Assay

Mitochondrial activity and by extension cell viability was determined by the established MTT (3-[4–Dimethylthiazol-2-yl]-2,5-diphenyltetrazolium bromide) chromogenic assay [31]. 3T3-L1 pre-adipocytes were plated into 96-well plates in a seeding density of 5000 cells/well. Cells were treated with dilutions of 5% *v*/*v* and 10% *v*/*v* of the initial extracts in growth medium, for 24 h or 48 h. Each treatment was performed in 6 replicates. Immediately after treatment, 50 μL of MTT substrate (1 mg/mL, Sigma-Aldrich Chemie GmbH, Taufkirchen, Germany) were added in cells cultured in 100 μL growth medium per well, in the presence of extracts dilutions. After 3 h of incubation, formazan crystals were solubilized by the addition of 100 μL of dimethyl sulfoxide (DMSO) (AppliChem GmbH, Darmstadt, Germany). Formazan dye was quantified spectrophotometrically at 492 nm, using a microplate reader. Cell viability results were calculated as percentages of medium-only treated control. As negative control (complete cell death), cells were treated with 5 mM hydrogen peroxide (H_2_O_2_) (Sigma-Aldrich Chemie GmbH, Taufkirchen, Germany).

### 2.5. Differentiation into Beige Adipocytes

To induce the differentiation into mature beige adipocytes, supra-confluent 3T3-L1 pre-adipocytes cultured in growth medium were treated with dexamethasone (1 μΜ), insulin (1 μg/mL), T_3_ (2 nM), troglitazone (5 μΜ), indomethacin (750 nM) and 3-isobutyl-1-methylxanthine (IBMX, 0.5 mM) for 2 days (Day 1 to Day 3 of differentiation, “induction” period), followed by culture in growth medium supplemented with 1 μg/mL insulin (“maintenance” medium), until day 10, replacing the media every other day (all reagents were purchased from Sigma-Aldrich Chemie GmbH, Taufkirchen, Germany). During this 10-day differentiation protocol, extracts of CS, HTP aerosol, ECIG aerosol, ambient air and pure nicotine (Sigma-Aldrich Chemie GmbH, Taufkirchen, Germany)were administered in cells simultaneously with the differentiation inducers (i.e., every 2 days), starting on Day 1 of differentiation. The dilutions of extracts applied to cells were determined on the basis of the results of the cell viability assay (ensuring that cells would survive the exposure to all extracts) and adjusted to achieve the same final nicotine concentration of 3 μg/mL for all extracts. The dilution factor of ambient air extract was selected according to the dilution of CS extract. In order to identify the discrete effects of nicotine-alone in our experiment, cells were separately treated with 3 μg/mL of pure nicotine.

In a separate experiment, to ascertain the capacity of thus differentiated cells to express the typical thermogenesis-related gene *Ucp1*, on day 10, mature beige 3T3-L1 cells were treated with the adenylate cyclase agonist forskolin (10 μΜ,Sigma-Aldrich Chemie GmbH, Taufkirchen, Germany) for 4 h. The dose of forskolin was based on published data regarding the induction of *Ucp1* expression in beige adipocytes [25].

### 2.6. Oil Red O Staining

At the end of the 10 day-differentiation protocol, the amount of lipid accumulation of mature beige cells was analyzed using Oil-Red O staining. In brief, control and extract-treated cells were fixed in 4% formaldehyde (Sigma-Aldrich Chemie GmbH, Taufkirchen, Germany) and were then incubated with Oil Red O solution (Sigma-Aldrich Chemie GmbH, Taufkirchen, Germany) for 1 h. Staining solution was discarded and cells were washed with distilled water. Stained adipocytes were observed under a light microscope for cell and morphology (Leica DM IK LED FLUO, Leica Microsystems, Wetzlar, Germany). Representative pictures of the stained whole well monolayers were also obtained using a Zeiss Stemi DV4 dissecting microscope (Carl Zeiss Microscopy GmbH, Jena, Germany). Oil Red O staining was extracted using isopropanol (Sigma-Aldrich Chemie GmbH, Taufkirchen, Germany) and quantified by spectrophotometric analysis at 550 nm. For the estimate of relative lipid accumulation, data were normalized to the ambient air extract-treated cells.

### 2.7. RNA Isolation and Quantitative Real-Time PCR

Total RNA was isolated from cells, using NucleoSpin RNA kit (Macherey Nagel GmbH & Co. KG, Düren, Germany), according to manufacturer’s instructions. cDNA was synthesized from 1 μg of total RNA, using PrimeScript RT reagent kit (Takara Bio Inc., Shiga, Japan). Real-time PCR analysis was performed on the LightCycler^®^ 96 Instrument (Roche, Basal, Switzerland), using Kapa SYBR Fast qPCR Kit (Kapa Biosystems, Wilmington, MA, USA). Cycling conditions were as follows: 95 °C for 3 min, 40 cycles of amplification at 95 °C for 3 s and at 60 °C for 30 s, and a final melting curve analysis step. All samples were run in triplicate. Amplification efficiency was calculated for each primer pair using the standard curve method. Relative gene expression was determined using the ΔΔCt method. *Ribosomal Protein S18* (*Rps18*) was selected as the reference gene and data were normalized to the ambient air extract. 

The primer sequences for gene expression analysis, are shown at Table 1. Primers were designed using the publicly available Primer-Blast designing tool [32].

### 2.8. Statistical Analysis

Statistical analysis was performed using one-way ANOVA with post-hoc least significant difference (LSD) test for multiple comparison. All results are expressed as mean ± standard error of the mean (SEM) of 3 independent experiments. Statistical significance was defined as * *p* < 0.05 or ** *p* < 0.01.

## 3. Results

In the current study, we aimed to investigate if the exposure of 3T3-L1 pre-adipocytes to cigarette and alternativesmoking/vapingproducts could affect their differentiation to mature beige adipocytes. For this purpose, we prepared aqueous extracts of CS, HTP and ECIG aerosol in cell growth medium, which were subsequently analyzed by LC/MS-MS, to identify the exact nicotine levels dissolved in the culture medium. Nicotine levels detected in each extract are displayed in Table 2. Based on previous research data [21], we used a different number of 1R6F cigarettes and HTP sticks for the generation of extracts, to achieve comparable nicotine levels, as verified by our results (87.35 μg/mL in the CS extract vs. 92.73 μg/mL in the HTP extract). Forty-eight puffs of ECIG liquid containing 1.2% *w*/*w* nicotine generated an extract with a significantly lower nicotine content, 51.50 μg/mL, while as expected, in the ambient air extract, only background levels of nicotine were detected. The determination of nicotine concentration in the extract-enriched media was used to ascertain the ability of the medium to capture components of the CS or the aerosols and served as a basis in order to determine the desirable dilution of each extract that would be used in cell cultures, ensuring that all extracts would be applied at equivalent nicotine concentrations as an equalizing factor.

**Figure 1:**
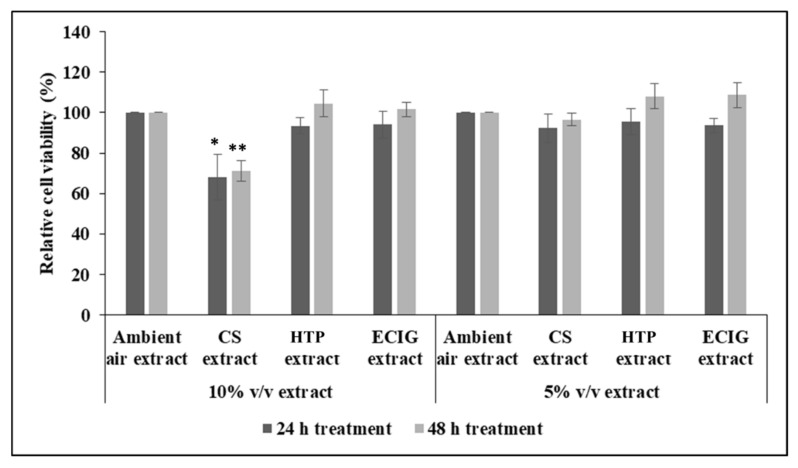
Effect of extracts on 3T3-L1 pre-adipocyte viability. The effect of extracts on metabolic activity and thus, viability of 3T3-L1 pre-adipocytes, after 24 h and 48 h of treatment, was assessed by the MTT-based chromogenic assay. Dilutions of 5% *v*/*v* and 10% *v*/*v* of the extracts were administered. Data are presented as the mean ± SEM of 3 independent experiments done in triplicate (*n* = 3). Statistical analysis was performed using one-way ANOVA with post-hoc LSD test for multiple comparisons. Statistical differences are indicated by * *p* < 0.05 and ** *p* < 0.01.

Initial experiments to measure cell survival after 24 h and 48 h of treatment with each extract, showed decrease in cell viability with the 10% *v*/*v* CS extract (cell survival of 68.04% at 24 h and 71.18% at 48 h), relative to the ambient air extract or relative to the other treatments, as well. Statistical analysis by one-way ANOVA revealed significant reduction of *p* = 0.041 and *p* = 0.003 for the 24 h and 48 h CS treatment, respectively. Further reducing the concentration of CS extract to 5% *v*/*v* had no effect on cell survival (Figure 1). The rest of the extracts did not impair cell survival at either 5% or 10% *v*/*v* (Figure 1).

Therefore, for the differentiation to mature beige adipocytes, concentrations of extracts lower than or equal to 5% *v*/*v* could be applied to the cells. To ensure equivalent nicotine concentrations in all extracts (3 μg/mL), treatment was performed with 3.33% *v*/*v* CS and HTP extracts and 5% *v*/*v* for the ECIG extract. Ambient air extract was also used at 3.33% *v*/*v*. To be able to attribute any effects, or lack thereof, to the presence or absence of nicotine, cells were also treated with 3 μg/mL of pure nicotine.

**Figure 2:**
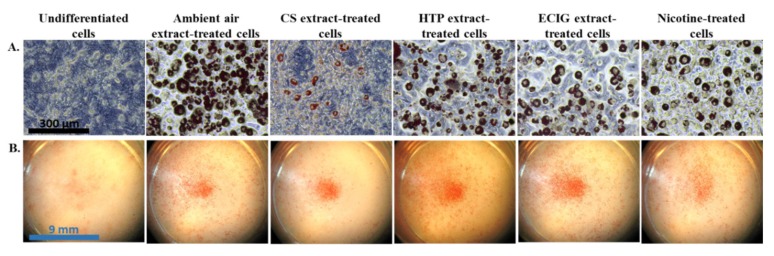
At the end of the differentiation process (10 days), cells were stained with Oil Red O. (**A**). Stained adipocytes were observed under the light microscope, revealing the lipid droplet formation in beige adipocytes. (**B**) Pictures of stained monolayers of adipocytes were obtained using a dissection microscope.

**Figure 3:**
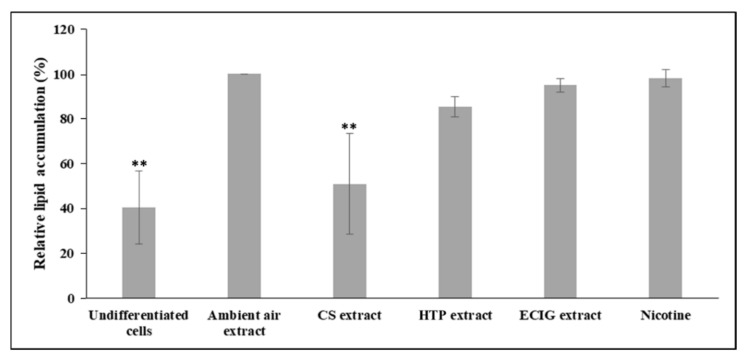
Effect of extracts on lipid content in beige adipocytes. 3T3-L1 pre-adipocytes were differentiated in the presence of each treatment for 10 days, whereupon cells were fixed and stained with Oil Red O. Undifferentiated 3T3-L1 cells maintained in parallel were also undergone the staining process. The extracted Oil Red O solution was quantified by spectrophotometric analysis at 550 nm. To determine relative lipid accumulation, data were normalized to the ambient air extract-treated cells. Histograms display the mean of the ratios ± SEM of 3 independent experiments (*n* = 3). Statistical analysis was performed using one-way ANOVA with post-hoc least significant difference (LSD) test for multiple comparison. Statistical differences are indicated by ** *p* < 0.01.

The effect of repeated exposure to extracts on the induction of differentiation of 3T3-L1 cells to beige adipocytes was evaluated by the morphological features and the expression of certain brown and beige-selective differentiation markers. Oil Red O staining confirmed the phenotype of beige adipocytes, as it revealed the formation of multilocular lipid droplets in the cytoplasm (Figure 2A). The observation of the stained cell monolayers under the dissection microscope showed substantial difference in the extent of differentiation (the proportion of differentiated to non-differentiated cells) in the cells exposed to the CS extract, compared to the ambient air extract and to the cells treated with the other extracts or pure nicotine (Figure 2B). Similarly, the following quantification of the extracted Oil Red O solution and the concomitant assessment of relative lipid accumulation displayed statistically significant reduction in cells differentiated in the presence of CS extract, relatively to the ambient air extract-treated cells (*p* = 0.004) and to the other treatments (*p* < 0.05) (Figure 3). A slight, non-significant decrease of lipid accumulation was also observed in the case of adipocytes exposed to the HTP extractrelative to ambient air extract (Figure 3).The absorbance values obtained in the case of undifferentiated cells are due to the remaining, non-specific staining of the wells and of the normal lipid content of the undifferentiated cells (i.e., in the absence of lipid droplets). 

**Figure 4:**
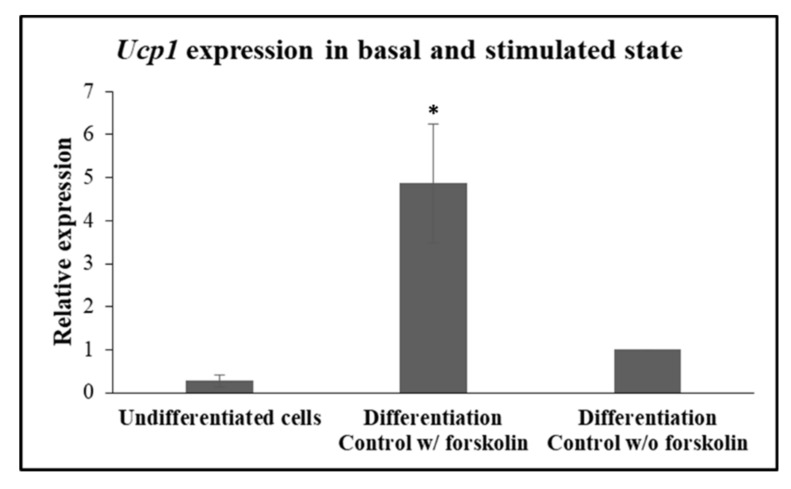
Expression of *Ucp1* in undifferentiated and differentiated 3T3-L1 cells, either unstimulated or treated with 10 μM forskolin, for 4 h prior to harvesting for RNA isolation. Values display the mean ± SEM of 3 independent experiments (*n* = 3). Statistical analysis was performed using one-way ANOVA. * *p* < 0.05.

In accordance with previously published data, induction of the expression of the obligate brown-beige marker, *Ucp1,* is regulated and rapidly responds to beta-adrenergic receptor stimulation and the ensuing cyclic adenosine monophosphate (cAMP) in beige adipocytes [25]. In accordance with this work, expression of *Ucp1* was detected in very low levels in undifferentiated cells and in 3T3-L1 differentiated to beige adipocytes that were not treated with forskolin (Figure 4). Indeed, when, on day 10, 3T3-L1 adipocytes were treated with 10 μM forskolin for a brief 4 h before harvesting for mRNA isolation, *Ucp1* expression was detected at significantly higher levels (*p* = 0.014), in comparison with the levels seen in forskolin-unstimulated cells (Figure 4), validating that the differentiated cells according to our protocol behave as *bona fide* “beige” adipocytes in our hands.

**Figure 5:**
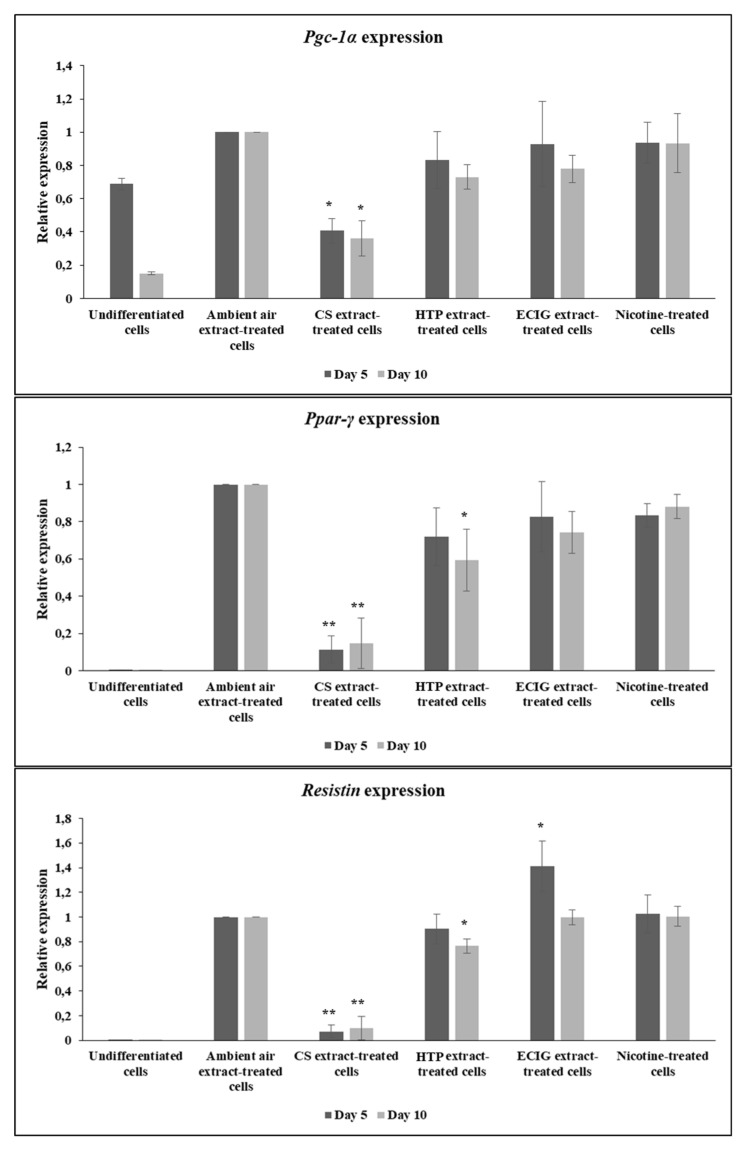
Expression of markers of adipogenic differentiation. At day 5 and day 10, total RNA was isolated and mRNA levels of *Pgc-1α*, *Ppar-γ* and *Resistin* were analyzed. Rps-18 was used as the reference gene (endogenous control) and data were normalized to the ambient air extract-treated cells. Values are mean ± SEM of 3 independent experiments (*n* = 3). Statistical analysis was performed using one-way ANOVA with post-hoc LSD correction. Statistical differences are indicated by * *p* < 0.05 or ** *p* < 0.01.

Subsequently, the potential of 3T3-L1 pre-adipocytes to differentiate under the effect of extracts was further evaluated by probing the expression at the mRNA level of brown- and beige-specific differentiation marker *Pgc-1α* and the general adipogenic markers *Ppar-γ* and *Resistin.* Expression analysis was assayed both at mid-differentiation, at Day 5 and at the end of differentiation (Day 10), to study potential differences occurring at various stages of the phenotypic differentiation. Consistent with the abovementioned results related to morphological observation and lipid accumulation, CS extract significantly decreased *Pgc-1α* mRNA levels, relatively to the ambient air extract (*p* = 0.02), ECIG extract (*p* = 0.035) and nicotine (*p* = 0.033), at 5 days of differentiation (Figure 5). *Pgc-1α* expression was also significantly reduced following CS exposure at Day 10 of the differentiation process, compared to all the other treatments (*p* < 0.05). *Ppar-γ* and *Resistin* expression in CS extract – treated cells was also significantly lower, relatively to all other treatments, at both time points (*p* < 0.01 for *Ppar-γ* and *Resistin* expression, both at 5 and 10 days of differentiation) (Figure 5). Also, at 10 days of differentiation, HTP extract-treated cells exhibited significantly decreased *Ppar-γ* expression, compared to the ambient air extract-treated cells (*p* = 0.028) and decreased *Resistin* expression relatively to ECIG and nicotine-treated differentiating cells (*p* < 0.05) (Figure 5), although the expression of both genes was still much higher than cells treated with CS. It is noteworthy that nicotine-treated cells displayed similar levels of expression of adipogenic markers with the ambient air extract-treated cells (Figure 5), suggesting that the effect of impaired differentiation observed is nicotine-independent. Finally, ECIG extract administration caused only a slight but significant increase in *Resistin* expression levels, at 5 days, compared to the ambient air extract (*p* = 0.045) (Figure 5).

## 4. Discussion

To the best of our knowledge, this is the first study to examine and compare the effects of CS, ECIG, and HTP on pre-adipocyte differentiation towards a beige adipocyte fate. CS was found to be more cytotoxic in 3T3-L1 pre-adipocytes, while adverse effects of CS were also observed on adipogenic differentiation. In contrast, limited such effects were observed when cells were exposed to HTP aerosol, confined to *Ppar-γ* and *Resistin* expression, and no effects similar to those seen by CS were observed from exposure to ECIG aerosol. The activity observed with the CS extract-enriched medium cannot be attributed to nicotine, since equivalent nicotine exposure from the other products and, even more, exposure to pure nicotine did not result in similar effects.

It has been well established that CS enhances lipolysis in the whole vertebrate organism in an indirect way, through nicotine-induced release of catecholamines, epinephrine, and norepinephrine, which bind on the membrane β-adrenergic receptor of adipocytes [33]. The expression of nicotinic acetylcholine receptors (nAChRs) on the adipocyte surface [34,35] and the decrease in the lipolytic effects of nicotine in subcutaneous adipose tissue upon the nAChR blockade suggest the presence of a second, direct mechanism of action of nicotine on adipocytes [33]. Further studies have revealed that the a7nAChR mediates nicotine-stimulated activation of adenosine monophosphate AMP-activated protein kinase α2 (AMPKa2) in adipocytes by increasing reactive oxygen species (ROS) levels [10]. Adipose AMPKα2 activation leads to inhibition of fatty acid synthase and consequently, of lipogenesis, resulting in body weight reduction, but it also impairs insulin signaling which, in combination with the elevated levels of circulating free fatty acids, contribute to the development of tissue insulin resistance [10,11]. Animal studies have reported that maternal and offspring CS exposure during lactation is responsible for obesity development in adulthood [36]. Similarly, intrauterine nicotine exposure caused an increase in epididymal white adipose tissue and hypertrophy of adipocytes with increased *Ppar-γ* expression, leading to metabolic syndrome in the progeny [37]. These studies overwhelmingly focused on white adipose tissue. In contrast, the extent of nicotine- or cigarette smoke-elicited effects on brown or “beige” adipocytes are still very limited. In this respect, recent research has shown that neonatal CS exposure evoked decreased thermogenic capacity of brown fat at an adult age [38]. Nicotine treatment of 3T3-L1 pre-adipocytes exhibited a complex effect depending on which stage of differentiation towards beige adipocytes occurred the exposure; at the early differentiation stage, the beige-like phenotype was impaired, whereas it was enhanced when nicotine was administered only at the later, “maintenance” stage or during the entire differentiation process [39]. It was suggested that the increased levels of ROS and oxidative stress markers induced by nicotine exposure [10] contribute to the differentiation of 3T3-L1 pre-adipocytes to mature adipocytes [40]. In all, it is still largely unclear what is the effect of CS, nicotine and, even more, the newer methods of self-administration of nicotine (HTP, ECIG), on the ability of pre-adipocytes to differentiate towards a “brown/brite/beige” adipocyte phenotype. This is important, because this type of cell has recently received a lot of attention, because induction of “brownization” and/or maintenance of a beige/brown adipocyte function seems to ameliorate multiple metabolic indexes, among others blood glucose levels, tissue sensitivity to insulin and cardiovascular response to an atherogenic diet [41,42,43,44,45].

For these reasons, we decided to test and compare, for the first time, the effect of media enriched with CS, HTP and ECIG on the differentiation of a well-studied pre-adipocytic cell line, 3T3-L1, towards beige adipocytes.

In this study, the administration of diluted extracts to 3T3-L1 pre-adipocytes showed that cell viability was only reduced by CS extract in a dose- and time-dependent manner. Following cell survival experiments and nicotine determination in the extracts, 3T3-L1 cells were treated with concentrations of extracts that, according to our data, do not to affect cell viability and growth, containing 3 μg/mL nicotine, during the entire differentiation process to mature beige adipocytes (during both the “induction” [Day 1–3] and “maintenance” [Day 3–10] periods). This was an important step to ensure that cells would survive the exposure. The use of nicotine as an exposure normalization measure for all extracts is representative of realistic use by humans who are expected to use different products in such a way as to obtain an individually-targeted similar nicotine intake [17]. Our results demonstrate that the diluted CS extract significantly impaired the induction of differentiation towards a beige adipocyte fate, as proven by the decreased amount of mature differentiated cells characterized by multilocular lipid droplets. This finding was also in agreement with the reduced mRNA levels of the brown- and beige-selective marker *Pgc-1α* and of the general adipogenic markers *Ppar-γ* and *Resistin*. Although *Resistin* is widely considered to be a white adipocyte-specific marker, it is also expressed in brown adipose tissue, though at lower levels [46]. Of great interest is that the effect of CS extract on differentiation is not attributed to nicotine and is probably due to other CS compound(s) that could attenuate differentiation. 3T3-L1 adipocytes have indeed been shown to express nAChRs and respond to nicotine concentrations within the range that were used herein [10].The inability of pure nicotine to affect markers of differentiation (relative to ambient air extract) indicated that nicotine by itself had no impact at the levels used in these experiments. This is in agreement with a previous study finding no effect of exposure to nicotine on lipid accumulation in beige adipocytes [39].This reduced differentiation potential caused by CS that we observed in our study is consistent with in vitro studies of osteogenic differentiation of mesenchymal stem cells (MSCs) [47,48]. Indeed, Shaito and co-workers showed that both CS and ECIG extracts prevented osteogenic differentiation from progressing [47]. The very low *Ucp1* expression in cells not stimulated with forskolin is in line with previous studies [25]. The fact that treatment with forskolin resulted in elevated *Ucp1* expression, validates the differentiation procedure used, verifying that undifferentiated cells were, indeed, transformed to beige adipocytes capable of responding to a sympathetic signal in short order (4 h). In contrast to CS-enriched medium, HTP, and ECIG extract-enriched media did not significantly affect the induction of differentiation to beige adipocytes, despite the fact that they delivered to the cells nicotine exposure levels equivalent to CS. Collectively, these findings suggest that there are different metabolic effects of HTP and ECIG compared to CS, and these effects are independent of nicotine. Smoking is associated with the development of metabolic syndrome and smoking cessation reduces this risk [49,50,51].Whether and to what extend HTP and ECIG differ from conventional cigarettes in this respect is still unknown and further preclinical evidence in a whole organism, corroborated by clinical data, is needed to probe this.

In this study, extracts were generated using glassware impingers to trap cigarette smoke and aerosol from HTP and ECIG. This is an established method that has been used previously in in vitro studies of cytotoxicity of both tobacco cigarettes and alternative smoking/vaping products [52,53,54]. Moreover, quantitative detection of aldehydes in ECIG aerosols has been effectively performed using the impinger trapping system [15]. Alternative exposure methods involve the collection of particulate matter using Cambridge filters or whole smoke/vapor cell exposure by air-liquid interface culture, a type of culture, usually, applied to airway epithelial cells to simulate human lung conditions. The advantage of extract production through impingers is that it captures constituents of both particulate and gas phases of smoke, but the exact composition of the derived extract is at this moment unknown. Therefore, the chemical composition of extracts is definitely dependent on the capture system of the emissions. It has been shown previously that different ECIG aerosol capture systems, that is, impingers and Cambridge filter pads, have different efficiencies and that various compounds present in the ECIG emissions cannot be trapped using the filter pad method and vice versa [55]. In our study, the collection buffer was the culture medium, thus, water-soluble constituents are expected to be more prevalent. In contrast, the use of filters would trap particulate phase only, while extraction from the filter would require different organic solvents, potentially incompatible with cell survival and differentiation, especially upon repeated applications, as required here. The topographic characteristics of smoking/puffing regime that we followed are generally considered representative of human smoking behavior [56] and have been used in ECIG aerosol analysis studies [57]. As a measure of dose-adjustment among the different extracts, we performed LC/MS-MS analysis on extracts to quantify the levels of dissolved nicotine. 

Some limitations are applicable to this study. We did not use nAChR blockers to further investigate the effects of nicotine on cell differentiation from exposure to different products. This is because pure nicotine treatment at the onset and during differentiation was no different compared to treatment with ambient air extract (control). It is possible that exposure to nicotine, after differentiation has been initiated, may alter the differentiation and metabolic function of adipocytes after differentiation and can be investigated in a future study. While nicotine was excluded as the causative factor for the adverse effects of tobacco CS on cell differentiation, it would take a totally different study and logistics to identify specific compounds and mechanisms causing the adverse effectsobserved. CS contains more than 7000 chemicals, many of which are known toxins or carcinogens [1,2], and it is probably the combined effects of several chemicals in the mixture that finally impact adipocyte survival and differentiation.The cell line used in this study (3T3-L1) has been extensively used for decades as an informative model to probe adipocyte biology, and has been instrumental in advancing our understanding of the adipocyte differentiation process [58,59]. However, further studies using different cell lines, such as cultures of primary adipocytes, are needed to expand our understanding of the effects of tobacco cigarettes and alternative nicotine products on the mechanisms underlying adipocyte differentiation and adipogenesis. Finally, while in vitro studies are valuable in identifying pathophysiological mechanisms, their clinical relevance needs to be ultimately confirmed through epidemiological studies. It is currently unknown whether the differences between products observed in this study can be translated to differences at a clinical level.It should be emphasized that while the present study examined aspects of the effects of these products on metabolically important cells, their overall safety and risk profile in other systems is an important determinant of their hypothesized potential to serve as harm reduction products. Still, a recent review of the evidence by National Academies of Sciences, Engineering and Medicine concluded that ECIG aerosol contains fewer numbers and lower levels of most toxicants than does smoke from combustible tobacco cigarettes [60], while a review by Public Health England stated that, compared with cigarettes, heated tobacco products are likely to expose users and bystanders to lower levels of particulate matter and harmful and potentially harmful compounds [61]. In vitro studies have also shown potentially adverse biological effects of these products [62,63].However, some clinical studies in smokers switching to ECIGs have reported improvement in physiologic parameters such as endothelial function [64] and respiratory function in asthmatics [65].Therefore, the global consensus is that a lot more research is needed to accurately quantify the harm reduction potential of these nicotine delivery products relative to tobacco cigarettes, as well as the effects of their use by non-smokers.

In conclusion, significant differences in cytotoxicity and pre-adipocyte differentiation to beige adipocytes were found between different nicotine products, at equivalent nicotine content; CS exhibits severe adverse cellular effects on both cell survival and the ability to differentiate, while HTP and ECIG exhibit limited or no adverse effects. These effects cannot be attributed to the presence of nicotine in the media. It is now well-established that smoking is a risk factor for the development of the metabolic syndrome, via multiple intersecting mechanisms [11,12,66]. Our study indicates that detrimental effects of CS on beige adipocytes may, in some extent, contribute to this. The effect of CS is not imitated, in our experimental setup, by HTPs and ECIGs. Therefore, HTPs and ECIGs seem more compatible than CS with brown/beige adipocyte differentiation and functionality, both of which are known to overall ameliorate metabolic function. A logical extension of this observation is that the preservation of brown/beige adipocyte function by HTPs and ECIGs may provide another reason to differentiate these products from tobacco cigarettes. However, further preclinical studies and, ultimately, clinical and epidemiological studies are necessary to firmly establish whether these products can indeed cause “less harm” in comparison to cigarette smoking.

## Figures and Tables

**Table 1:** Sequences used for gene expression analysis.

Gene	Primer Sequence (5’→3’)
*Rps18*	F: GCCATGTCTCTAGTGATCCC
	R: GAGGTCGATGTCTGCTTTCC
*Ucp1*	F: ACTGCCACACCTCCAGTCATT
	R: CTTTGCCTCACTCAGGATTGG
*Pgc-1α*>	F: AGCCGTGACCACTGACAACGAG
	R: GCTGCATGGTTCTGAGTGCTAAG
*Pparg*	F: GATGCACTGCCTATGAGCACTT
	R: AGAGGTCCACAGAGCTGATTCC
*Resistin*	F: AAACAAGACTTCAACTCCCTG
	R: TTTCTTCACGAATGTCCCACG

Ribosomal Protein S18 (*Rps-18*), Uncoupling protein 1 (*Ucp-1*), Peroxisome proliferative activated receptor, gamma, coactivator 1 alpha (*Ppargc1α* or *Pgc-1α*), Peroxisome proliferator activated receptor gamma (*Pparg*).

**Table 2:** Determination of nicotine levels detected in extract-enriched media, using liquid chromatography–tandem mass spectrometry (LC–MS/MS). Data are presented as the mean ± standard deviation (SD) (*n* = 6 technical replicates in 2 independent repeats).

Type of Extract	Nicotine Concentration (μg/mL)
CS extract	87.35 ± 3.65
HTP extract	92.73 ± 3.08
ΕCIG extract	51.50 ± 1.29
Ambient air extract	1.04 ± 0.08
